# A Gapless, Unambiguous Genome Sequence of the Enterohemorrhagic *Escherichia coli* O157:H7 Strain EDL933

**DOI:** 10.1128/genomeA.00821-14

**Published:** 2014-08-14

**Authors:** Haythem Latif, Howard J. Li, Pep Charusanti, Bernhard Ø. Palsson, Ramy K. Aziz

**Affiliations:** a Bioengineering Department, University of California San Diego, La Jolla, California, USA; b Department of Microbiology and Immunology, Faculty of Pharmacy, Cairo University, Cairo, Egypt

## Abstract

*Escherichia coli* EDL933 is the prototypic strain for enterohemorrhagic *E. coli* serotype O157:H7, associated with deadly food-borne outbreaks. Because the publicly available sequence of the EDL933 genome has gaps and >6,000 ambiguous base calls, we here present an updated high-quality, unambiguous genome sequence with no assembly gaps.

## GENOME ANNOUNCEMENT

*Escherichia coli* serotype O157: H7, a causative agent in food poisoning outbreaks leading to hemorrhagic colitis or hemolytic uremic syndrome, gained public attention following its association with an outbreak in 1993 related to the U.S. fast-food chain Jack-in-the-Box (1, 2) and another large outbreak among schoolchildren in Sakai, Japan in 1996 (3). Moreover, *E. coli* O157:H7 strains are known for their prophage-rich genomes and are currently considered the bacterial genomes with the largest number of integrated phages (4, 5). Strain EDL933 (ATCC 43895), isolated from ground beef linked to a massive hamburger outbreak in Michigan, USA in 1982 (6), is the prototypic reference strain representing this pathotype.

Although the full genome of EDL933 was sequenced and published in 2001 (5), the deposited assembled genome has >6,000 ambiguous base calls and a chromosomal gap of 4,000 bp. While the utility of this reference genome, cited in >3,200 publications, is indisputable, several analyses reliant on a pristine reference (e.g., single nucleotide polymorphism studies) are hindered by those ambiguities and gaps. EDL933 has long phage-associated repeat regions >7 kb. Microbial genomes with these characteristics are the most complex to assemble (7), so we resorted to single-molecule sequencing using PacBio followed by polishing using Illumina short-reads to complete the EDL933 sequence. This produced a gapless genome assembly, with no ambiguous base calls, and an updated genome annotation.

Genomic DNA from the EDL933 strain was prepared for PacBio and Illumina sequencing. PacBio libraries were prepared according to standard library preparation procedures with Blue Pippen size selection for >20-kb fragments and sequenced using P5/C3 chemistry and 3 h movies on the RS II system at the UCSD Genomics Core, San Diego, CA. Illumina libraries were prepared according to the TruSeq DNA PCR-Free sample preparation kit protocol (Illumina) and paired-end sequenced (2×250) on a MiSeq. SMRTAnalaysis 2.2.0 HGAP v2 assembly of PacBio reads (66,927) produced three polished contigs: two corresponding to the chromosome and one the plasmid. When compared with the reference, NC_002655, a region of high read density within the two chromosomal contigs was shown to be a large duplication that unites the two contigs. After the plasmid and chromosome were circularized, reads were mapped back to the assembled sequences to check for variants by first using Bridge Mapper (RS_BridgeMapper.1) with PacBio reads and then Breseq v0.24rc6 (8) with Illumina short reads. Coverage was ~100× for PacBio data and ~300 for Illumina data. The final assembled genome was automatically annotated, then manually corrected, through the RAST server using SEED annotation tools (9, 10).

The updated EDL933 genome consists of a 5,547,323-bp chromosome and a 92,076-bp plasmid compared with 5,528,445 and 92,077 bp in the current EDL933 assembly. This gapless assembly eliminates 6,641 ambiguous base calls in the current EDL933 chromosome including 2,413 non-N ambiguous bases and 4,000 N’s belonging to a chromosomal gap. The updated genome has 5,675 and 97 annotated coding sequences (CDSs) compared with 5,286 and 99 CDSs found in the current reference chromosome and plasmid, respectively.

### Nucleotide sequence accession numbers.

This whole-genome shotgun project has been deposited in GenBank under the accession numbers CP008957 and CP008958. The RAST version is available at http://rast.nmpdr.org under job 157998.
